# The NRF2, Thioredoxin, and Glutathione System in Tumorigenesis and Anticancer Therapies

**DOI:** 10.3390/antiox9111151

**Published:** 2020-11-19

**Authors:** Morana Jaganjac, Lidija Milkovic, Suzana Borovic Sunjic, Neven Zarkovic

**Affiliations:** Laboratory for Oxidative Stress, Division of Molecular Medicine, Rudjer Boskovic Institute, Bijenicka 54, 10000 Zagreb, Croatia; morana.jaganjac@irb.hr (M.J.); lidija.milkovic@irb.hr (L.M.); borovic@irb.hr (S.B.S.)

**Keywords:** cancer, reactive oxygen species (ROS), antioxidant mechanisms, NRF2 (nuclear factor erythroid 2 like 2) pathway, thioredoxin (TRX) system, glutathione (GSH) system, anticancer therapy

## Abstract

Cancer remains an elusive, highly complex disease and a global burden. Constant change by acquired mutations and metabolic reprogramming contribute to the high inter- and intratumor heterogeneity of malignant cells, their selective growth advantage, and their resistance to anticancer therapies. In the modern era of integrative biomedicine, realizing that a personalized approach could benefit therapy treatments and patients’ prognosis, we should focus on cancer-driving advantageous modifications. Namely, reactive oxygen species (ROS), known to act as regulators of cellular metabolism and growth, exhibit both negative and positive activities, as do antioxidants with potential anticancer effects. Such complexity of oxidative homeostasis is sometimes overseen in the case of studies evaluating the effects of potential anticancer antioxidants. While cancer cells often produce more ROS due to their increased growth-favoring demands, numerous conventional anticancer therapies exploit this feature to ensure selective cancer cell death triggered by excessive ROS levels, also causing serious side effects. The activation of the cellular NRF2 (nuclear factor erythroid 2 like 2) pathway and induction of cytoprotective genes accompanies an increase in ROS levels. A plethora of specific targets, including those involved in thioredoxin (TRX) and glutathione (GSH) systems, are activated by NRF2. In this paper, we briefly review preclinical research findings on the interrelated roles of the NRF2 pathway and TRX and GSH systems, with focus given to clinical findings and their relevance in carcinogenesis and anticancer treatments.

## 1. Introduction

According to the World Health Organization, cancer is the second leading cause of death, accounting for 9.56 million deaths and an incidence of 18.1 million new cases in 2018, with the GLOBOCAN estimation of 29.5 million new cases and 16.4 million deaths by 2040 [1]. Consequently, cancer remains a global burden and an elusive, ever-changing disease with an extremely complex biology. The high inter- and intratumor heterogeneity is governed by cancer’s need to grow and spread in the organism while surviving therapy-induced unfavorable conditions. Genetic alterations inducing survival pathways combined with metabolic reprogramming ensure the action of diverse players contributing to tumor development and progression.

Reactive oxygen species (ROS) are recognized as essential players contributing to tumorigenesis or suppressing it, with an important role in anticancer therapy as well. Although previously perceived as exogenously and endogenously derived byproducts of cellular metabolism with signaling abilities that affect cellular functions in a concentration-dependent manner, the perception of ROS has been upgraded since. Nowadays, they are considered as cellular tools that are, upon certain stimuli, purposely produced by the cell in an amount that will elicit a specific feedback reaction within the cell or in neighboring ones [2]. The tuning of ROS levels is intertwined with metabolism and antioxidative machinery, as in normal cells and cancer cells, too. However, cancer cells often have more ROS than normal ones, a feature that is exploited by most conventional chemo- and radiotherapy.

The nuclear factor, erythroid 2 like 2 (NRF2) pathway is the main pathway activated upon ROS production. Its activation induces the expression of over 250 genes, regulating diverse processes from redox homeostasis to detoxification, carbohydrate and lipid metabolism, autophagy, apoptosis, DNA repair, etc. [3]. The antioxidant mechanisms involved in ROS balancing, such as thioredoxin (TRX) and glutathione (GSH) systems, not only protect from carcinogenesis but can support it as well. A recent clinical study on colorectal cancer patients demonstrated that redox biomarkers could have a role in the infiltration of inflammatory cells and tumor budding [4]. The same study also demonstrated that the levels of oxidative stress parameters vary based on the stage of cancer. In addition, GSH and TRX are both depicted as important players in carcinogenesis. While GSH is required for cancer initiation, TRX is a predominant mechanism governing cancer progression by abolishing detrimental ROS levels in already established neoplasm [5]. Noteworthy, the NRF2 pathway is often activated in various types of cancer, emphasizing its dual role in tumorigenesis.

Only the understanding of all intertwined cancer-related components, particularly the contributors and/or suppressors of cancer growth, will bring us closer to better-targeted therapy. This review will explore the importance of the NRF2 pathway and TRX and GSH systems in carcinogenesis in relation to anticancer treatment strategies.

## 2. Reactive Oxygen Species—Friend or Foe?

ROS are continuously formed in aerobic organisms as byproducts of normal intra/intercellular metabolism or in response to adequate stimuli, as is the case of inflammation. Depending on the ROS type, reactivity, and their diffusion distance, their effect on macromolecules varies [6]. Thus, superoxide anion (O_2_^●−^) ROS are generated by the action of complex I and complex III via an electron transport chain in mitochondria [7], while membrane-bound nicotinamide adenine dinucleotide phosphate (NADPH) oxidase and lipoxygenases also contribute to O_2_^●−^. Additionally, huge amounts of ROS, in particular O_2_^●−^, are formed during phagocytosis [6]. The dismutation of O_2_^●−^ occurs spontaneously or enzymatically, with superoxide dismutase (SOD) generating hydrogen peroxide (H_2_O_2_), which is a more stable non-radical form of ROS with a greater diffusion distance that may therefore affect targets distant from the initial oxidative insult. At low concentrations, ROS have important signaling roles both intra- and intercellularly; however, at higher concentrations they have toxic effects. Likewise, H_2_O_2_ is an important metabolic signal for insulin secretion in β-cells [8], however excessive H_2_O_2_ affects mitochondria and causes β-cell dysfunction [9,10]. Similarly, ROS generated during the respiratory burst of granulocytes can have dual roles in carcinogenesis [6]. Although an important anticancer effect of granulocytes was demonstrated in vitro [11,12], the in vivo results can be contradictory. In experimental animal tumor models, it was shown that ROS can reverse the process of tumor development, leading to spontaneous tumor regression [13,14], and can decelerate tumor growth, extending the survival of animals [15]. Opposite to that, it is known that ROS can also promote tumor development [13,14], so one also needs to consider the importance of intercellular redox signaling in tumor development. Hence, it was recently shown that also hypochlorous acid intercellular redox signaling is involved in granulocyte-mediated tumor inhibition [16].

One of the key mechanisms by which ROS achieve their biological effects is the well-documented induction of either reversible or irreversible modifications of proteins, altering their structure and cellular functions. For example, the oxidative modification of erythrocyte spectrin alpha chain, band 3 protein, and glyceraldehyde-3-phosphate dehydrogenase can affect the erythrocyte structure and energy metabolism [17]. Similarly, the oxidation of mitochondrial adenosine triphosphate synthase beta subunit can affect the enzyme catalytic activities and potentially have a detrimental role for neurons [18].

Both O_2_^●−^ and H_2_O_2_ can serve as precursors for highly reactive hydroxyl radicals (^●^OH) that can induce direct oxidative damage to macromolecules. Transition metals, such as iron, can promote ROS and support tumorigenesis [19,20]. O_2_^●−^ and H_2_O_2_ are accessible to iron via the Fenton reaction, converting them to ^●^OH, and can contribute to the initiation of ferroptosis [21], which is also considered in anticancer approaches as a potential target for oncotherapy [22].

On the other hand, ROS can also damage DNA, causing strand breaks or oxidative DNA lesions. As an example, the ^●^OH-induced nucleic acid oxidative damage can yield a variety of base lesions, among which 8-oxo-2′deoxyguanosine (8-OHdG) and 8-oxo-guanosine have received the most attention [23,24]. The accumulation of unrepaired or defectively repaired DNA damage could eventually promote various disorders, among which cancer is the prominent disease.

Moreover, the bis-allylic site of polyunsaturated fatty acids is highly susceptible to ROS induced damage. The peroxidation of lipids is profoundly triggered by ^●^OH and hydroperoxyl radical, yielding as final products reactive aldehydes, among which is the most potent “second messenger of ROS”, 4-hydroxynonenal (4-HNE). 4-HNE was first discovered fifty years ago and was thought to be a toxic product of lipid peroxidation [25]. Since then, a huge amount of effort has been put into investigating the roles of 4-HNE, and today we know that, depending on its location and concentration, it can act as a double-edged sword, exhibiting pathological or desirable physiological effects. The high reactivity of 4-HNE is due to its three functional groups: hydroxyl group, carbonyl group, and C=C double bond [26]. 4-HNE can act either directly or by forming adducts with macromolecules, such as proteins [27] or nucleic acids [28,29]. Consequently, 4-HNE can modulate various cellular functions of non-malignant as well as cancer cells [30,31,32,33] and can play a role in tumorigenesis [34,35,36].

## 3. Redox Homeostasis

Excessive ROS generation alters the cellular redox homeostasis built by antioxidant defense systems to cope with the negative effects of ROS. On the other side, endogenous antioxidant defense systems are affected by the supplementation of exogenous antioxidants, which is common in both healthy and ill people.

The major endogenous antioxidants responsible for cellular ROS detoxification are the TRX system, the GSH system, peroxiredoxin, SOD, and catalase (CAT). The removal of O_2_^●−^ by SOD elevates cellular H_2_O_2_ that can act as a molecular mediator of various signaling pathways, such as insulin, NRF2, and the c-Jun N-terminal kinase (JNK) signaling pathway [10,37]. Whether H_2_O_2_ will promote cell survival or induce apoptosis depends on its concentration. Thus, cells have several mechanisms for H_2_O_2_ detoxification. Catalase, peroxiredoxin, and the TRX and GSH systems all promote the conversion of H_2_O_2_ to H_2_O and molecular oxygen. Although these might be considered as major cellular antioxidant defenses, one should bear in mind that other antioxidants—for example, protein deglycase DJ-1—can also determine the fate of the tumor, interact with NRF2, regulate GSH levels, and promote TRX (reviewed in [38]).

Therefore, alterations in redox homeostasis are of high importance for cancer development. Although ROS have an important role in the initiation and promotion of tumor development, they are also crucial factors that can inhibit tumor viability and growth, so tumor cells modify their own antioxidant network to enable them to escape the anticancer effects of ROS. Among endogenous antioxidants in the current review, special attention is given to the NRF2, TRX, and GSH antioxidant defense mechanisms in tumorigenesis.

### 3.1. The NRF2 Pathway in Tumorigenesis

**Figure 1:**
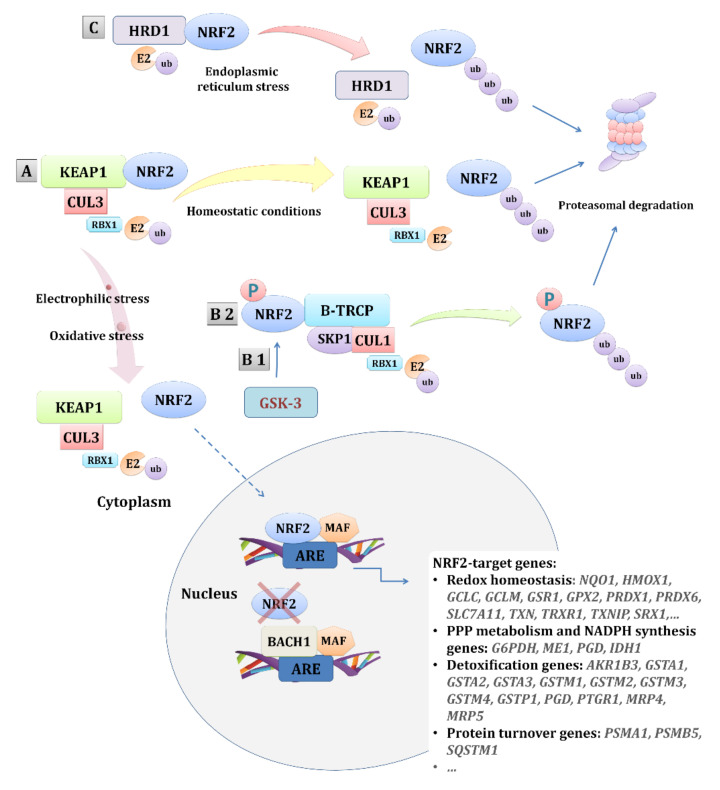
Regulation of the NRF2 pathway. NRF2 is regulated by three E3 ubiquitin ligases. The main regulation is by the complex KEAP1-CUL3-RBX1 (**A**). Additionally, NRF2 can be regulated by the β-TRCP-SKP1-CUL1-RBX1 complex in a GSK-3-dependant (**B**1) or -independent (**B**2) manner. Upon endoplasmic reticulum stress, NRF2 can also be regulated by HRD1 (**C**). The main regulation (**A**) involves the repression of NRF2 by KEAP1 and its proteasomal degradation in basal conditions, while, under oxidative stress conditions, the NRF2-KEAP1 binding is disrupted, leading to NRF2 nuclear translocation and the activation of its target genes. Other mechanisms of NRF2 repression involve the β-TRCP-SKP1-CUL1-RBX1 complex (**B** 1/2) that, through binding with the Neh6 domain of NRF2, particularly enhanced by the GSK-3 phosphorylation of NRF2, or, by the binding of endoplasmic reticulum stress-induced HRD1 (**C**) with the Neh4-5 domains of NRF2, mediate its degradation. In addition, other proteins, such as BACH1, can negatively regulate the transcriptional activation of the NRF2-target genes by competing with NRF2. Abbreviations: AKR1B3—aldo-keto reductase family 1 member B; ARE—antioxidant response element; BACH1—BTB and CNC homolog 1; β-TRCP—β-transducin repeat-containing protein; CUL1—cullin 1; CUL3—cullin 3; E2—ubiquitin-conjugating enzyme 2; G6PDH—glucose-6-phosphate dehydrogenase; GCLC—glutamate-cysteine ligase, catalytic subunit; GCLM—glutamate-cysteine ligase, modifier subunit; GPX2—glutathione peroxidase 2; GSR1—glutathione reductase 1; GSTA—glutathione *S*-transferase alpha; GSTM—glutathione *S*-transferase mu; GSTP—glutathione *S*-transferase pi; HMOX-1—heme oxygenase 1; HRD1—E3 ubiquitin-protein ligase synoviolin, SYVN1; IDH1—isocitrate dehydrogenase 1; KEAP1—Kelch-like ECH-associated protein 1; MAF—musculoaponeurotic fibrosarcoma; ME1—malic enzyme 1; MRP—multidrug resistance-associated proteins; NQO1—NAD(P)H quinone dehydrogenase 1; NRF2—nuclear factor, erythroid 2 like 2; PGD—phosphogluconate dehydrogenase; PRDX—peroxiredoxin; PSMA1—proteasome 20S subunit alpha 1; PSMB5—proteasome 20S subunit beta 5; PTGR1—prostaglandin reductase 1; RBX1—ring-box 1; SKP1—S-phase kinase-associated protein 1; SLC7A11—solute carrier family 7 member 11; SQSTM1—sequestosome 1.; SRX1—sulfiredoxin 1; TRXR1—thioredoxin reductase 1; TXN—thioredoxin; TXNIP—thioredoxin interacting protein; ub—ubiquitin.

The NRF2 pathway is the main pathway activated as a response to oxidative stress. In homeostasis, transcription factor NRF2, encoded by the *NFE2L2* gene (nuclear factor, erythroid 2 like 2) is mainly under the control of its repressor, Kelch-like ECH-associated protein 1 (KEAP1), and subjected to ubiquitination and proteasomal degradation [39]. Additionally, NRF2 can also be repressed by β-transducin repeat-containing protein (β-TRCP) in a glycogen synthase kinase-3 (GSK-3) -dependent and -independent manner [40] or in an HRD1 (E3 ubiquitin-protein ligase synoviolin, *SYVN1*) -dependent manner upon endoplasmic reticulum (ER) stress [41] (Figure 1). Aside from the mentioned mechanisms of NRF2 regulation, numerous proteins and molecules interact with either KEAP1 or NRF2 or even compete with NRF2, such as BTB and CNC homolog 1 (BACH1) [42], contributing to the highly complex regulation/activation of NRF2, the feature particularly accentuated in cancer (reviewed in [43,44,45,46,47]).

Current research recognizes the dual role of NRF2 in tumorigenesis. NRF2 was first perceived as a tumor suppressor due to its role in reducing damaging ROS and environmental carcinogens, thus protecting cells from potential neoplastic transformation. Mice *Nrf2*-knockout models have pointed out the importance of the NRF2 pathway activation in the suppression of carcinogen-induced cancer initiation (e.g., in the skin [48], breast [49], and forestomach [50]). In humans, an NRF2 single nucleotide polymorphism (SNP) homozygous allele (-617A7A) exhibits a decreased expression of NRF2 and its target cytoprotective products, and is consequently associated with an increased risk of lung cancer in smokers [51]. In addition, the genetic variants in *NFE2L2,* NAD(P)H quinone dehydrogenase 1 (*NQO1*), nitric oxide synthase 3 (*NOS3*), and heme oxygenase 1 (*HMOX-1*), exhibit lower ROS detoxification capabilities—although they are not associated with a breast cancer risk individually—in postmenopausal women with three or more of such modifications, increasing the risk of breast cancer, especially when combined with a high iron intake [52].

Conversely, the discovery of the hyperactivation of NRF2 in quite a number of tumors, conferring an advantage to tumor cells and resulting in growth promotion and therapy resistance, revealed its new role as an oncogene. In this context, the constitutive activation of NRF2 contributes not only to the progression and chemoresistance in the already-established tumor cells but also to the tumor development itself. Indeed, the constitutive activation of NRF2, associated with an increase in cellular antioxidant enzymes and a diminution in the ROS levels, contributes to arsenite-induced human bronchial epithelial cell transformation [53].

Genetic alterations (e.g., *NFE2L2* gain-of-function mutations, *KEAP1* loss-of-function mutations, and co-occurring driver mutations), interactions with different proteins interfering with the NRF2-KEAP1 bond, and epigenetic and posttranslational modifications can all induce NRF2 activation. Emerging data imply the varying involvement of the NRF2 pathway among tumors (some are listed in Table 1). Cancers with NRF2 hyperactivating mutations share a set of upregulated target genes, including key regulators involved in the thioredoxin and glutathione system, metabolic enzymes, transporters, and others, enabling a cancer growth advantage and consequently a worse overall survival. The upregulation of these genes by NRF2 highly depends upon a more assessable chromatin environment of antioxidant responsive elements (AREs) [54].

In lung cancer, the co-occurring mutations in *KEAP1*, *NFE2L2*, or cullin *3 (CUL3*) observed in 7% of non-small cell lung cancer (NSCLC) patients with *EGFR* (Epidermal Growth Factor Receptor) mutations indicate the activation of the NRF2 pathway as an acquired mechanism, leading to the resistance to usually beneficial EGFR-tyrosine kinase inhibitor therapy [55]. Another protective mechanism observed in lung cancers is metabolic reprogramming to glutaminolysis, mostly observed in *KRAS* (Kirsten rat sarcoma viral oncogene homolog)-mutant lung adenocarcinoma, which exerts a loss of *STK11*/LKB1 (serine/threonine kinase 11, liver kinase B1) and co-occurring *KEAP1* mutation [56]. In addition, the NRF2 molecular signature, including genes important in activating TRX and GSH systems such as glutathione peroxidase (GPX) 3 and thioredoxin-like 1, is suggested as an excellent predictor of cancer remission and overall survival in patients with lung cancer [57]. The growth advantage and resistance to therapy in NSCLC patients with a worse prognosis are associated with the activation of the NRF2 pathway, contributing to a higher expression of multidrug-resistant protein-3 [58].

The other mechanisms of NRF2 activation include diverse proteins that compete with NRF2 in binding with KEAP1. Examples of these are proteins with an ETGE motif, such as dipeptidyl peptidase 3 protein [59], cell cycle-related kinase (CDK20), as well as others. The overexpression of CDK20 in lung cancer leads to tumor progression and resistance to radio and chemotherapies [60]. The p62 is another NRF2-binding competitor of KEAP1. Its upregulation was suggested as a protection mechanism of hepatocellular carcinoma (HCC)-initiating cells in the oxidative stress hostile environment that promotes HCC carcinogenesis [61], as well as anticancer drug tolerance in tumor regions positive for the hepatitis C virus [62]. The alkylation of KEAP1 by succinylacetone [63] or the hypermethylation of the KEAP1 promoter region may lead to NRF2 activation. For instance, the tumor-specific hypermethylation of the KEAP1 promoter region was suggested as a specific feature of a clear cell renal carcinoma [64].

Crosstalk between the NRF2 pathway and other proteins can contribute to carcinogenesis and therapy resistance. Hence, it was shown that the progression of HCC requires metabolic changes involving TRAP1 and NRF2 as an early event, leading to an expression pattern of glucose-6-phosphate dehydrogenase that correlates with grading, metastasis, and poor prognosis [65]. Another example of this is the overexpression of TRIM25 (Tripartite motif-containing protein 25) and NRF2, associated with the protection of HCC cells upon ER-induced ROS [66]. The association of BACH1 and NRF2 was shown to promote lung cancer metastasis. Mechanistically, the mutations in *KEAP1* found in lung adenocarcinoma patients lead to the activation of the NRF2 pathway and an increase in HMOX-1. Since the degradation of BACH1 by F-box only protein 22 (FBOXO22) necessities heme, the NRF2-induced HMOX-1 reduces heme levels, thus stabilizing BACH1 and promoting the transcription of the pro-metastatic genes. The antioxidant supplementation of *N*-acetyl cysteine and vitamin E mimics the NRF2-HMOX-1 action, leading to the BACH1 stabilization and glycolysis induction in *KRAS*-mutant lung cancer. Therefore, lung adenocarcinoma patients with a high BACH1 signature are associated with increased metastasis and worsened survival [67,68]. Conversely, FBXO22 promotes carcinogenesis in colorectal cancer [69] and HCC [70] by degrading PTEN (phosphatidylinositol 3,4,5-trisphosphate 3-phosphatase and dual-specificity protein phosphatase) and p21, respectively, while revealing both roles (protumorigenic and antimetastatic) in breast cancer [71].

### 3.2. The Thioredoxin System and Thioredoxin-Domain-Containing Protein Family in Tumorigenesis

**Figure 2:**
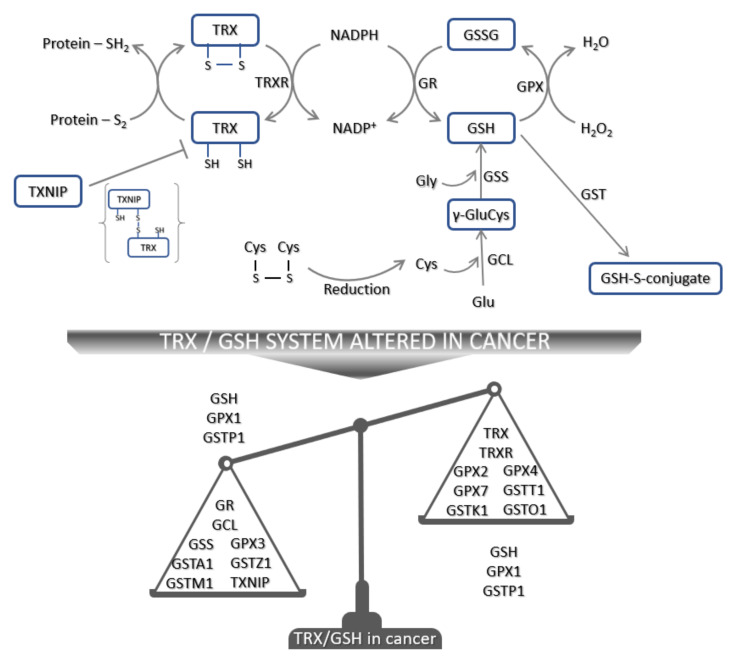
Schematic presentation of TRX and GSH antioxidant defense mechanisms. Oxidized TRX is reduced to the active reduced form of TRX by TRXR that uses NADPH as the main electron source. Reduced TRX reduces oxidized substrates. However, this can be inhibited by TXNIP, resulting in TXNIP-TRX complex formation. Moreover, extracellular cystine once imported in the cell can be reduced by the TRX system and used for GSH synthesis. GCL catalyzes the formation of γ-GluCys from Cys and Glu. Further addition of Gly by GSS produces GSH. During the detoxification of ROS, GSH is converted to GSSG and its recycling depends on GR and NADPH. In addition, during the detoxification of other molecules—e.g., 4-HNE—GSH conjugation by GST is the main step. In cancer, TRX and/or GSH systems are altered and their expression in cancer is schematically presented. Abbreviations: Cys—cysteine, γ-GluCys—gamma glutamylcysteine, GCL—glutamate cysteine ligase, Glu—glutamine, Gly—glycine, GSH—glutathione, GSS—glutathione synthetase, GPX—glutathione peroxidase, GR—glutathione reductase, GSH—reduced glutathione, GSSG—oxidized glutathione, GST—glutathione *S*-transferase, NADP^+^—oxidized NADPH, NADPH—nicotinamide adenine dinucleotide phosphate, TXNIP—thioredoxin interacting protein, TRX—thioredoxin, TRXR—thioredoxin reductase.

The thioredoxin system, one of the key regulators of cellular redox homeostasis, comprises TRX, thioredoxin reductase (TRXR), nicotinamide adenine dinucleotide phosphate (NADPH), and thioredoxin interacting protein (TXNIP) (Figure 2). The TRX system requires NADPH to reduce the oxidized form of TRX. Reduced TRX is needed for the recycling of oxidized thioredoxin peroxidase/peroxiredoxin [139]. The family of thioredoxin-domain containing proteins (TXNC) are also redox regulators, and today 17 members of the TXNDC family are known [140]. When an oxidized substrate is reduced by the action of Cys32 and Cys35 of the reduced dithiol form of TRX, oxidized disulfide TRX is generated. Oxidized TRX is converted back to an active reduced TRX by TRXR at the expense of NADPH [141]. Thus, an adequate supply of NADPH and TRXR activity is crucial for this process.

In the last few decades, numerous studies have investigated the role of the TRX system and some members of the TXNDC protein family in tumorigenesis (Table 2). The overexpression and hyperactivation of cytoplasmic TRXR (TRXR1) have been reported for various cancer types, such as brain cancer [142], breast cancer [143], HCC [144], lung cancer [144,145], oral [146,147], and tongue squamous cell carcinoma [148] (Table 2). Mitochondrial TRXR (TRXR2) was also found to be upregulated in tumor tissue [149]. Moreover, tumor cells overexpress TRX [143,148,150,151,152,153] to cope with excessive ROS, and its expression is closely related to the pathological grade of the tumor [154,155,156,157,158]. Additionally, the downregulation of TXNIP, a TRX inhibitor, will result in a decrease in TXNIP-TRX complexes, protecting cells from the effects of excessive ROS and resistance to therapy. Indeed, it is common for various types of cancers to downregulate TXNIP [159,160]. The TXNIP was suggested to be used as a prognostic marker as its expression inversely correlates with the pathological grade of tumor [142,161], while its overexpression can indicate a longer survival of cancer patients [156,162,163,164]. Among the TXNDC protein family, TXNDC5 has been studied the most and was found to be overexpressed in tumor tissues [165,166,167], and its high expression correlates with poor survival [168]. Other TXNDCs reported to be altered in tumors are TXNDC9 [169,170] and TXNDC17 [171].

In addition to the important role of the TRX system and TXNDCs as guardians of cellular redox homeostasis, they also modulate various cellular pathways that might affect tumor development (Table 3), and some of the mechanisms are described below. TRX, mainly located in the cytosol and frequently referred to as cytoplasmic TRX (TRX1), interacts with different proteins in order to maintain cellular redox homeostasis, while in the state of oxidative stress it translocates to the nucleus modulating the activity of transcription factors. The TRX1 induces nitric oxide synthase type 3 and the *S*-nitrosylation of death receptor CD95, modulating apoptosis [187]. Nitric oxide synthase 2 overexpression induces the *S*-nitrosylation of mitochondrial TRX (TRX2) and caspase 3, altering their activity and promoting tumor growth [188]. TRX1 induces S100P, leading to an additional increase in TRX1 through a positive feedback mechanism via the upregulation of phosphorylated ERK1/2 and the downregulation of TXNIP [155]. TRX1 overexpression decreases tumor suppressor PTEN [157,189] and causes an increase in phosphorylated AKT (protein kinase B) that can consequently induce S100A4 and promote epithelial to mesenchymal transition (EMT), migration, and invasion of tumor cells [190]. Recently identified mitochondrial TRXR isoform (TRXR3) was found to reduce TRX2 and promote tumor cell survival [191]. On the contrary, the inhibition of TRXR alters the mitochondrial membrane, reduces tumor growth, and induces apoptosis [149,192,193]. Several microRNAs (miR) are known to inhibit TRXR, such as miR-125b-5p [194], miR-124 [195], and miR-17-3p [196]. Similarly to TRX, TRXR can be inactivated by nitrosylation [197], while its acetylation increases catalytic activity [198].

Furthermore, metabolic and oxidative stress as well as hypoxia or hyperglycemia can promote TXNIP expression [199,200,201], which is also considered as a tumor suppressor [161]. The overexpression of TXNIP induces mitochondrial ROS generation, activates MAPK [159], and promotes apoptosis and cell cycle arrest [160,163]. Thus, the downregulation of TXNIP is frequent in tumor cells, and some of the mechanisms by which that is accomplished include targeting the TXNIP N-terminus [202], affecting the TXNIP promoter [177,203], or binding to the 3′-untranslated region (3′-UTR) of TXNIP [204,205]. The inhibition of TXNIP promotes tumor cell proliferation, EMT, and metastasis [204,205,206]. However, TXNIP can be upregulated by inhibiting histone deacetylases [207,208], bromodomain and extra-terminal domain [209], phosphatidylinositol-3-kinase (PI3K)/AKT pathway [210], the downregulation of HER1/2 [163], or focal adhesion kinase [211]. The poor survival of triple-negative breast cancer patients correlates with elevated c-MYC and decreased expression of TXNIP, which is probably due to c-MYC binding to the TXNIP promoter [177]. The secretome of monocyte-derived foam cells contains 4-HNE and was shown to increase the TXNIP expression of endothelial cells [212], which could be attributed to 4-HNE’s ability to inhibit the expression of c-MYC [213].

Among the TXNDCs, TXNDC5 can have a role in both tumor progression and tumor suppression. The inactivation or downregulation of nuclear receptor 4A1 (NR4A1) downregulates TXNDC5, isocitrate dehydrogenase 1, and the mTOR (mammalian target of rapamycin) pathway, further promoting ROS generation, inducing apoptosis, and inhibiting tumor growth [214,215,216]. The downregulation of TXNDC5 was also reported to inhibit angiogenesis [217]. On the other hand, hypoxia-induced TXNDC5 via hypoxia inducible factor 1α (HIF1α) promotes tumorigenesis [166,218].

### 3.3. The Glutathione System in Tumorigenesis

The glutathione system, another key regulator of cellular redox homeostasis, comprises glutamate-cysteine ligase (GCL), glutathione synthetase (GSS), reduced glutathione (GSH), oxidized glutathione (GSSG, glutathione disulfide), GPX, glutathione reductase (GR), NADPH, and glutathione *S*-transferase (GST) (Figure 2). GSH is ubiquitously distributed within the cell and the availability and level of GSH depend on its synthesis by GCL and GSS, the recycling of GSSG by GR and NADPH, and its detoxification activity via GST-mediated conjugation to molecules. GST belongs to phase II metabolism, and today seven classes of GST are known [232]. The detoxification role of GSH and GST is crucial to enable cells to cope with various stressors. However, alterations in GSH systems can promote tumorigenesis (Table 4). Decreased blood GSH was seen in cancer patients [233,234]. Interestingly, the GSH level was found to be increased in head and neck carcinoma [234] while the opposite was reported for colorectal cancer [235]. Moreover, cancer patients frequently have a decreased blood GSH and GPX activity [233,236,237,238,239]. Remarkably, the expression of GPX in tumor tissue depends on the GPX isoform. In that regard, GPX1, GPX3, and GPX7 expression were found to be decreased [144,240,241,242,243] in tumor tissues, while the expression of GPX2 and GPX4 was found to be upregulated [144,146,244,245,246,247]. In addition, GSTA1, GSTM1, and GSTZ1 are reported to be downregulated in tumor tissue and can correlate with a poor prognosis [63,248,249,250,251], while GSTT1, GSTO1, and GSTK1 are mostly reported to be upregulated in tumor tissue compared to the normal surrounding tissue [252,253]. The expression level of GSTP1 in tumor tissue is controversial [144,253,254,255], and its involvement in tumorigenesis could at least in part depend on its hypermethylation [256,257,258,259,260].

Tumor cells tend to modulate the GSH system in order to survive (Table 5), and some mechanisms are described below. The upregulation of the NRF2 pathway promotes GCL, yielding elevated GSH and promoting tumorigenesis [110,272]. In the case that GSH is depleted, tumor cells overcome protein homeostasis by deubiquitinases [273]. Thus, the combined inhibition of deubiquitinases and GSH generation would be needed for malignant destruction [273]. The methylation of GPX1 promotor downregulates GPX1 [274], which can induce the activation of the AKT/GSK-3β/SNAIL pathway, promoting EMT [243]. GPX2 overexpression is also implicated in EMT [245]. However, miR-17-3p can inhibit GPX2, altering mitochondrial respiration and consequently rendering tumor cells susceptible to anticancer therapy [196]. Several other miRs also modulate the GSH system. Thus, miR-196a targets GPX3 [242], affecting GPX3-mediated cell death and promoting tumorigenesis [275]. Additionally, miR-133b downregulates GSTP1 by targeting GSTP1 3′-UTR [276]. GSTP1 is also downregulated by the methylation of the CpG island [277], while the recruitment of early B cell factor 1 to its promoter upregulates GSTP1 [255]. The overexpression of GSTP1 can induce cell cycle arrest [254], as well as GPX4 deficiency [278], and can thus be used as potential targets in anticancer therapy. GSTPs catalytic activity varies depending on the target. For example, GSTA4-4 has the highest activity towards 4-HNE and also the lowest rate with respect to 4-HNE adduction compared to others [279]. The conjugation of 4-HNE with GSH is the major route for 4-HNE elimination from cells.

## 4. Modulation of Antioxidant Defense Systems in Anticancer Therapy

The perception that antioxidants can protect cells from detrimental levels of ROS has led to several large-scale studies with somewhat disappointing results. Data suggesting the beneficial effects of antioxidant supplementation are limited [287,288], while more show no effects or even imply that antioxidants can increase cancer risk [289]. Among these, a Finnish trial on the effect of vitamin E and beta carotene on the incidence of lung cancer and other cancers in male smokers raised a lot of skepticism against the possible beneficial effects of the fat-soluble vitamins, stressing mostly their negative effects, especially an increase in the lung cancer incidence [290]. However, the authors of the trial have overseen the fact that their treatment resulted in a several-fold overload of the supplemented antioxidants in plasma, indicating their even higher potential overload in the lungs. In the case of persistent oxidative stress, as is the one occurring in heavy smokers, these antioxidants, especially beta-carotene, could be oxidized into multiple toxic, mutagenic, and likely carcinogenic products, although at the same time beta-carotene could further maintain its antioxidant capacity [291], in particular for the lung cells. Similarly, the Alpha-tocopherol, beta-carotene cancer prevention (ATBC) study in 90′ linked the supplementation of beta-carotene with the increased risk of lung cancer among smokers [292], while the more recent findings attenuated so negative results showing that supplementary alfa-tocopherol and beta-carotene have no late effects on cancer incidence. Thus, after two decades, the authors of the ATBC trial revealed the preventive effect of moderate-dose alpha-tocopherol on prostate cancer that continued for several years after the trial, reducing prostate cancer mortality [293]. However, the issue of the use of antioxidants as adjuvant therapy is still under debate favoring the laconic “no use” approach [294,295], while the increased recurrence and mortality of breast cancer in patients who concurrently took dietary supplements with radio- and chemotherapy is alarming [296].

While considering the possible benefits or risks of using antioxidants in oncology, one should recall the pathophysiology of oxidative stress, which is aware of beneficial roles played by endogenous antioxidants, especially in the case of acute stress response (such as those induced by irradiation, toxic compounds, or ischemia/reperfusion), for which optimal interplay between water-resistant and water-soluble antioxidants is essential. If that is not the case, many antioxidants (in particular, those water-resistant—i.e., lipid-soluble—present in biomembranes) could become free radicals, even more harmful than the initial ROS that started oxidative stress. That is even more relevant for chronic oxidative stress, as occurs in cancer and inflammation, while the long-lasting or excessive use of exogenous antioxidants even bears a risk of uncontrolled interference with endogenous antioxidants, which is the main reason why antioxidants cannot be considered as panacea. Similarly, even in the case of mild and acute oxidative stress (like in exercise) the use “preventive” exogeneous antioxidants can block desirable hormetic feedback effects of exercise, that can cause a rise in the endogenous antioxidant capacities, mostly affecting NRF2, which may eventually increase the resistance of oxidative-stress associated disorders.

The NRF2 pathway is the main cellular defense mechanism activated upon exposure to oxidative stress, electrophilic stress, and xenobiotics. It regulates the expression of a great variety of cytoprotective genes, enabling cells to withstand unfavorable conditions and restore the homeostatic state. In an effort to reduce cancer incidence, past research has been focused on the activation of what was considered as a cancer-protective mechanism, the NRF2 pathway. Thus, the extensive search for activators of the NRF2 pathways begun. Many candidates emerged, some reaching clinical trials. Sulforaphane is one such candidate abundantly present in broccoli sprouts. Clinical trials with broccoli sprout extracts revealed the attenuation of cancer risk in individuals exposed to aflatoxins and air-borne toxins [297] and insufficient, although safe, anticancer activity in prostate cancer patients [298], while others are still ongoing (extensively reviewed in [299]). Curcumin is another NRF2 activator with pleiotropic activity [300]. To date, clinical trials investigating the effectiveness of curcumin treatment in diverse cancers revealed the omission of the expected effect. Yet, curcumin was found to be well-tolerable and safe [301]. Other NRF2 activators enrolled in clinical trials include resveratrol, bardoxolone-methyl (CDDO-Me), oltipraz, RTA-408 (omaveloxolone), etc. (reviewed in [302]).

While the translation of in vitro and in vivo observed NRF2 activator-induced benefits to the clinic are still scarce, caution concerning their usage arises. The negative effect of NRF2 activation was observed in diabetic patients. Standard diabetic drugs, saxagliptin and sitagliptin, were shown to increase the risk of a metastatic spread in patients who already have cancer, although they did not enhance cancer incidence. The underlying mechanism included prolonged NRF2 activation [303]. Therefore, the use of NRF2 activators should be cautiously evaluated, particularly in cancer patients, considering the hyperactivation of the NRF2 pathway observed in the vast majority of cancers.

The constitutive activation of NRF2, observed in many cancers, causes research to rethink the new possibilities for treating cancer that can amend acquired resistance to conventional therapy. Inhibitors of the NRF2 activation, and its target products such as TRX and GSH, have become a focus of this research. All-trans retinoic acid (ATRA), clobetasol propionate (CP), and apigenin are some of the examples of the NRF2 inhibitors under clinical investigation, while others show promise in cellular and animal models, including ARE expression modulator 1 (AEM1), ML385, 1-(2-cyclohexylethoxy)aniline (IM3829), malabaricone-A (MAL-A), etc. [39,47]. ATRA forms a complex with retinoic acid receptors (RARs) that bind with NRF2, thus interfering with the binding of NRF2 to ARE sequences and blocking the activation of the NRF2 pathway [304]. Although already in use for treating acute promyelocytic leukemia patients, its applicability in the treatment of solid tumors is still ambiguous and heavily ongoing (reviewed in [305]). CP is another NRF2 inhibitor currently evaluated in phase 2 clinical trials (NCT02368886) in patients with refractory metastatic colorectal cancer. CP prevents nuclear accumulation and promotes the β-TRCP-dependent degradation of NRF2 in a glucocorticoid receptor- and a GSK-3-dependent manner [306]. While apigenin was shown to inhibit NRF2 activation [307], further research revealed its multiple modes of action [308]. Apigenin did reach the clinical trial (NCT00609310) investigating the prevention of neoplasia recurrence, yet the study has been suspended. Auranofin, an FDA-approved drug for the treatment of rheumatoid arthritis, and buthionine sulfoximine (BSO), an inhibitor of GSH synthesis, have been in consideration as anticancer agents as well. While BSO failed in the clinic, auranofin is still under investigation in several clinical trials [309]. Its mode of action includes the inhibition of the activity of TRXR and thus the disruption of the TRX system [310].

The recent opinion suggests combinational therapy to be more adequate in cancer treatment because anticancer agents usually affect different pathways, not offering cancer cells to adapt so quickly as in monotherapy and is thus reducing the occurrence of chemoresistance. In addition, the mutual effect lowers the effective dose, which in turn attenuates the unwanted side effects of some drugs [311]. PI3K/AKT inhibitors, such as MK2206 (Merck), have shown some promise in clinical trials. Yet, in vitro and in vivo studies have revealed the non-responsiveness of such monotherapy in NSCLC, suggesting combinational therapy as an advantageous strategy. Thus, a synthetic lethality induced by MK2206 and TXNRD1 inhibitor auranofin, found to be dependent on the genetic status of KEAP1, shows promise in KEAP1 wild-type over mutant [312].

A more personalized approach, such as the analysis of patients’ mutational status and putting them in context with the known mechanisms of the anticancer treatments, could improve patients’ outcomes. For example, combining clinical data and in vivo and in vitro approaches revealed the importance of *KEAP1* and *NFE2L2* mutations in lung adenocarcinoma, correlating them with advanced stages and worse survival. Associating these mutations with *KRAS* mutations revealed the high dependence on glutaminolysis of *KRAS/KEAP1* or *KRAS/NFE2L2* mutants, a potentially exploitable feature in future therapy [313]. In addition, *KRAS/KEAP1* mutants were found to arise from a bronchiolar cell of origin and keep the pentose phosphate pathway active, another possible exploitable feature in therapy [314]. Moreover, in lung adenocarcinoma tumors co-occurring *KEAP1* mutations and STK11/LKB1 loss lead to metabolic reprogramming (glutamine metabolism), activating the pentose phosphate pathway and the tricarboxylic acid (TCA) cycle to maintain redox balance, suggesting a glutaminase inhibitor as a possible treatment strategy [56]. Xu et al. investigated the relationship between *NFE2L2/KEAP1* mutations, tumor mutational burden (TMB), and programmed death ligand 1 (PD-L1) expression. They found that *NFE2L2/KEAP1* mutations are present in various cancers, with the highest incidence found in lung squamous cell carcinoma. These mutations were linked with higher TMB and PD-L1 expression. Since the *NFE2L2/KEAP1* mutations in cancer are often associated with poorer overall survival, a survival analysis of NSCLC patients receiving immunotherapy revealed improved clinical outcomes in comparison to other treatments, suggesting its possible beneficial use for patients with mutations in *NFE2L2/KEAP1* [315]. In addition, *NFE2L2/KEAP1* mutations cause the constitutive activation of the NRF2 pathway and enhanced ARE activity, a feature suggested as being exploitable for a cancer suicide gene therapy. Leinonen et al. used a lentiviral vector expressing herpes simplex virus thymidine kinase (HSV-TK/GCV) under the regulation of ARE. They evaluated this approach in human lung adenocarcinoma cells. They showed this approach to be effective in both in vitro and in vivo and suggested it as a promising treatment in conjunction with traditional therapies [316,317]. Table 6 contains a summary of antioxidant defense system modulators and strategies in cancer incidence and therapy.

## 5. Conclusions

Cancer biology is a very complex process that includes the multifaceted interplay between antioxidant systems and ROS in determining cancer development, progression, metastasis, and regression. Such complexity is sometimes overseen while evaluating the effects of potential anticancer antioxidants, as in the ATBC trial in the 1990s. In the modern era, realizing that a more personalized and integrative biomedical approach could give more benefits for the prevention and therapy of cancer, we should focus on altered oxidative homeostasis in cancer cells.

Therefore, the relevance of the NRF2 pathway and TRX and GSH systems in carcinogenesis and in anticancer therapies has been extensively investigated. Targeting only one system can be beneficial, while the combined modulation of multiple antioxidant systems can give better anticancer results. Besides various synthetic agents, miRs should also be considered in anticancer therapies, as they have been shown to have an important role in the modulation of the NRF2 pathway and TRX and GSH antioxidant system efficiencies.

## Figures and Tables

**Table 1:** The involvement of the NRF2 pathway in diverse types of tumors.

Cancer Type	The NRF2 Pathway Activities	Ref.
Blood cancers	High expression of NRF2 in AML patients is governed by NF-κB and leads to chemoresistance.	[72]
	Nuclear accumulation of NRF2; lower nuclear levels of BACH1; and a higher expression of HMOX-1, NQO1, GCLM, and GSR were found to be protective mechanisms of bortezomib-resistant AML patients.	[73]
	IHC expression of NRF2 in bone marrow correlates with a risk of myelodysplastic syndromes and with the worse overall survival of high-risk patients.	[74]
	Higher NRF2 and HMOX-1 levels are found in the peripheral blood mononuclear cells of CLL patients vs. healthy donors.	[75]
	NF-κB-dependent activation of P62 activates the NRF2 pathway, ensuring resistance to ROS-inducible therapy in ROR1-high CLL patients.	[76]
	IHC expression of NRF2 and KEAP1 was higher in patients with diffuse large B-cell lymphoma than with reactive lymph node hyperplasia and rises with the tumor stage.	[77]
	Combination of NRF2, NRF1, and KEAP1 localized expression (high nuclear NRF2, high cytoplasmic and low nuclear NRF1, and low cytoplasmic KEAP1) is linked to worse overall survival in patients with diffuse large B cell lymphoma.	[78]
Lung cancer	Heterogenic distribution of *KEAP1* and *NFE2L2* mutations among NSCLC patients (with a frequency of 11.3% and NFE2L2 3.5%, respectively) is linked to chemotherapy resistance.In 80% of patients, co-occurrence with other cancer-related mutations was observed.	[79]
	*KEAP1/NFE2L2* mutations in metastatic lung adenocarcinoma are linked with a chemotherapy-resistant subtype and more progressive disease.	[80]
	NSCLC patients with brain metastasis have mutations in the KEAP1-NRF2-ARE pathway that provide a survival advantage and dissemination of circulating tumor cells.	[81]
	Higher protein expression of NRF2, peroxiredoxin, TRX, and sulfiredoxin in lung cancer tissues in comparison to the paired normal lung tissue implies their protective role against oxidative injury and compensation due to the high mitochondrial metabolism.	[82]
	In Japanese patients with lung cancer, *NFE2L2* mutations were mainly found in males with advanced stages of squamous cell carcinoma and worse overall survival.	[83]
	The most frequent co-mutations found within the NSCLC patients with the *KRAS* mutations include *KEAP1/NFE2L2* (27%). These co-mutations are a negative prognostic factor, predicting shorter survival and response to therapy.	[84]
	*KEAP1/ NFE2L2* mutation status predicts the risk of local recurrence after radiotherapy in NSCLC patients.	[85]
	NRF2 overexpression and lower expression of Beclin 1 are associated with worse prognosis in NSCLC patients. Higher expression of NRF2 was linked to a larger tumor, more advanced TNM stage, lymph node, and distant metastasis.	[86]
	*NFE2L2* mutations were observed more frequently in the advanced stages of NSCLC, particularly squamous cell carcinoma in the Japanese cohort.	[87]
	Early-stage squamous cell carcinoma is enriched with several somatic mutations, including mutually exclusive *NFE2L2* and *KEAP1*. Patients with *NFE2L2* mutations, especially co-occurring with *TP53* mutations, were linked with worse recurrence-free survival, while *KEAP1* and *TP53* co-mutants were associated with a poor response to adjuvant therapy.	[88]
	Somatic alterations of *NFE2L2*, *KEAP1*, or *CUL3* upregulate a specific set of 28 genes used to discriminate SCC in subgroups with an active NRF2 pathway and WT. SCC patients with the expression signature of an active NFR2 pathway have shown reduced benefit from adjuvant cisplatin/vinorelbine therapy.	[89]
	NAMS comprised of 50 tumor-associated genes can be used as an independent prognostic marker of recurrence-free survival and overall survival, with NAMS+ patients having a worse prognosis.	[57]
	Enrichment with *KEAP1* mutations and NRF2 overexpression is observed in more than 50% of TTF1-negative lung adenocarcinoma patients, who are known to have shorter survival.	[90]
	Nuclear expression of NRF2 was observed in 26% of NSCLC patients and more commonly seen in SCC than adenocarcinoma, while low or absent KEAP1 expression was detected in 56% of NSCLC and more commonly in adenocarcinoma. While nuclear NRF2 expression was associated with a worse overall survival in NSCLC and worse recurrence-free survival in SCC patients who underwent platinum-based adjuvant treatment, low or absent KEAP1 was linked with worse overall survival in SCC.	[91]
	Somatic mutations of the genes involved in oxidative stress response (*NFE2L2*, *KEAP1*) present in 21.1% of Chinese SCC patients. Frequent *NFE2L2*, *MAGEC1*, *NLRP3*, and *FAM5C* mutations were detected only in smokers.	[92]
	In 34% of SCC patients, there is the activation of the NRF2/KEAP1 pathway due to gene alterations in *NFE2L2*, *KEAP1*, *CUL*.	[93]
	Biallelic inactivation of *KEAP1* and activation of the NRF2 pathway (high nuclear and cytoplasmic staining of NRF2) is found in 41% of NSCLC tumors.	[94]
	*KEAP1* alterations and the overexpression of nuclear NRF2 were observed in 60% of true papillary adenocarcinoma.	[95]
	Higher NRF2, BCL-2, and BCL-XL mRNA levels observed in *TP53*-mutant NSCLC patients were linked with cisplatin-based chemotherapy resistance.	[96]
	*NFE2L2* mutations were observed predominantly in male smokers with SCC.	[97]
Breast cancer	Nuclear NRF2 predominantly in breast carcinoma cells observed in 44% of breast cancer patients was associated with worse recurrence and disease-free survival.	[98]
	Certain genetic polymorphisms in *KEAP1* can increase the risk of breast cancer and worsen patients’ survival, particularly when receiving adjuvant therapy.	[99]
	*NRF2* and *SRXN1* genetic polymorphisms could predict breast cancer risk and a survival outcome. For example, the *NRF2* rs2886162 AA genotype was associated with a worse survival, while the *NRF2* rs2706110 AA genotype was linked with an increased risk and the *SRXN1* rs6053666 C allele with a decreased risk of breast cancer.	[100]
	Low NRF2 mRNA expression levels were associated with worse disease-specific survival and overall survival, while higher levels of NRF2 mRNA in ER-positive tumors predict a better outcome. Comparison of the mRNA NRF2 expression levels in tumor vs. normal breast tissues revealed lower levels in tumors.	[101]
	GSTM1 * 1/0 genotype and genetic alterations in *KEAP1* and/or *NFE2L2* are linked with a worse prognosis.	[102]
	Out of 109 investigated SNPs related to oxidative stress genes, SNPs located in *NFE2L2*, metallothionein, *NQO1*, and peroxiredoxin 1 and 6 were associated with overall mortality.	[103]
	CXCL13-CXCR5 co-expression within breast tumors governed by high RelA conditions, low NRF2, and a lack of cxcr5 promoter DNA-methylation drive tumor progression and metastasis. NRF2 negatively regulates the transcription of *CXCL13*.	[104]
	NRF2 level decreased in the tumor in comparison to normal breast tissue. Lower NRF2 in the luminal B subtype is associated with a longer overall survival.	[105]
	The aggressive phenotype of breast cancer showing inverse expression of Caveolin-1 (low) and Mn-SOD (high) in tumor vs. normal tissue is associated with the activation of the NRF2 pathway, upregulation of Mn-SOD that leads to ROS production, and AMPK activation inducing glycolytic shift.	[106]
Esophageal cancer	Genetic alterations of *NFE2L2* are more common in ESCC (24%) vs. esophageal adenocarcinomas (1%).	[107]
	Enrichment of the NRF2-mediated oxidative stress pathway was suggested as a potential distinctive molecular mechanism of ESCC in African Americans.	[108]
	Genetic alterations of *NFE2L2* were one of the trunk mutations found in both precancerous lesions and ESCC, suggesting them to be early CNA events.	[109]
	High IHC expression of NRF2 was linked with metabolic reprogramming to glutathione synthesis and ROS detoxification and was associated with poor recurrence-free and overall survival in esophageal cancer patients.	[110]
	Evaluation of spatial intratumoral heterogeneity revealed *NFE2L2* and *KEAP1* mutations on branches, thus suggesting them as late events in ESCC.	[111]
	Comparison of ESCC in Asian and Caucasian patients identified *NFE2L2* as a race-biased gene, with a higher mutational rate in Asian patients.	[112]
	*NFE2L2* gain-of-function mutation occurred in 22% advanced ESCC and was linked with tumor recurrence and poor prognosis. Additionally, a molecular signature associated with *NFE2L2* mutation was linked with poor response to therapy and suggested as a potential prognostic marker to therapy.	[113]
	Somatic gene alterations of *NFE2L2* was found in 10% of ESCC. In addition, *NFE2L2*, *KEAP1*, and *CUL3* mutation*s* were shared among squamous cell carcinomas that originated from different parts of the body.	[114]
	NFE2L2 gene is significantly mutated in ESCC.	[115]
	Overexpression of *miR-432-3p* and negative relation with KEAP1 was observed in primary ESCC. Experimentally, *miR-432-3p* directly binds to the coding region of KEAP1, thus downregulating it and inducing the stabilization of NRF2.	[116]
Gastric cancer	NRF2 nuclear positivity was mostly present in cancer cells and associated with more aggressive tumors, worse overall survival, and resistance to 5FU-based adjuvant chemotherapy.	[117]
Pancreatic cancer	Nuclear NRF2 expression is associated with the expression of sulfiredoxin and predicts a worse survival in pancreatic adenocarcinoma.	[118]
Liver cancer	*NFE2L2* mutations were detected in 9.8% of hepatoblastoma, mainly in regions that are essential for binding with the KEAP1/CUL3 complex. Overexpression of *NFE2L2* target gene *NQO1* was the highest in *NFE2L2*-mutated tumors and was associates=d with metastasis, vascular invasion, and a worse outcome.	[119]
	Higher nuclear expression of NRF2 was observed in bigger tumors with poor differentiation and metastasis and was associated with a worse survival in HCC patients.	[120]
	Higher levels of NRF2 and 8-OHdG were observed in HCC cells. High 8-OHdG was associated with short survival. Experimentally, oxidative stress was suggested as a driver of HCC progression.	[121]
	mRNA expression of NRF2 and NRF2-related genes differs between HCC, adjacent tissue, normal liver, and liver diseases. Expression of NRF2 was the lowest in HCC and increased in cirrhosis and end-stage liver disease, while KEAP1 was higher in HCC vs. normal liver and increased in cirrhosis and end-stage disease. The expression of NQO1 was the highest in HCC and suggested as a possible biomarker of HCC.	[122]
	Out of 107 HCC samples, a high nuclear expression of NRF2 was observed in 75 samples. Expression of nuclear NRF2 and KEAP1 was inversely related and patients with high NRF2 and reduced KEAP1 had worse overall and disease-free survival. HCC patients with high NRF2 had a higher mRNA expression of AKR1B10, NQO1, and GCLM in tumor tissue.	[123]
	In HCC, the higher nuclear NRF2 observed in tumors vs. matched controls is linked with the increased production of PPP enzymes and the loss of aldolase A.	[124]
	*KEAP1* mutations were observed in 8% of HCC patients and linked with shorter disease-free survival.	[125]
	Overexpression of NRF2 and NQO1 was linked with tumor size, multiple intrahepatic recurrences, and poor prognosis.	[126]
	The upregulation of TRIM25 is correlated with a high NRF2 expression and low KEAP1 expression and predicts a poor prognosis in HCC patients.	[66]
Biliary tract cancer	*NFE2L2* is one of the significantly mutated gene in gallbladder carcinoma. Additionally, *KEAP1* and *NFE2L2* (exon 2 deletion) splice variants were also observed. KEAP1/NFE2L2 pathway activation was suggested as a significant prognostic predictor of survival.	[127]
	Higher NRF2 expression is associated with a worse overall survival in BTC patients receiving chemotherapy. SNPs located in *GPX4*, *CAT*, and *GSR* might modify chemotherapy effects on overall survival. Experimentally, the knockdown of GPX4, CAT, or GSR induced chemoresistance by increasing the ROS level and activating the NRF2-ABCG2 pathway.	[128]
Colorectal cancer	The expression of the proteins in the NRF2 pathway differs between cancer and normal tissue. Mean IHC density of KEAP1 and prohibitin was higher in tumor vs. normal tissue, with lower levels of NRF2, P62, and PARK7 than the distant normal tissue. The lowest level of KEAP1 and p21 was found in the adjacent normal tissue. NRF2 levels correlated with KEAP1 in the tumor and BACH1 in the normal tissue.	[129]
	A lower ratio of HMOX1/NRF2 mRNA level found in the tumor tissue of patients with distant metastasis might be used as a predictor of distant metastasis in CRC.	[130]
	Distinctive expression patterns of NRF2 and BACH1 were observed in CRC. While the increase in the NRF2 expression with the grade of malignancy did not contribute to the tumor invasiveness, the expression of BACH1 (the highest in normal mucosa, lower in adenoma, and again high in carcinoma) was associated with tumor invasiveness and metastasis.	[131]
Ovarian cancer	High cytoplasmic NRF2 was associated with low-grade histology and, together with high ERα expression, was associated with a better overall survival in patients with a serous cancer subtype.	[132]
Endometrial cancer	High nuclear NRF2 staining in 24.7% of EC mainly in *TP53/CNH*-like tumors (tumors with a mutation within the *TP53* coding sequence) and no nuclear staining in normal epithelial and stromal cells. No correlation between the nuclear NRF2 and mRNA levels of its target genes: *NQO1*, *GCLC*, *AKR1C3.* A subset of *TP53/CNH*-like tumors with a low mRNA NQO1 was associated with NRF2/TP53 cooperation that drives a more aggressive phenotype but initial better sensitivity to chemotherapy.	[133]
	NRF2 overexpression observed in ESC and its precancers might contribute to the worse overall prognosis in patients with ESC.	[134]
Head and neck cancer	Increased expression of NRF2 and to some extent thioredoxin was observed in head and neck squamous cell carcinomas, while KEAP1 overexpression was anatomic site-dependent and not negatively correlated with NRF2.	[135]
	Genetic alteration of the *KEAP1-NFE2L2-CUL3* axis in HNSCC induces the expression of genes, of which 17 selected are related to poor survival. They include genes associated with drug resistance, glutathione metabolism, oxidation-reduction processes, etc.	[136]
Skin cancer	mRNA and protein levels of NRF2 and NRF1 were the highest in benign naevi and decreased during melanoma carcinogenesis. High nuclear NRF2 or NRF1 expression in pigment cells was associated with a worse survival in patients without distant metastasis or without nodal metastasis, respectively.	[137]
	*NFE2L2* mutations were observed in 6.3% of skin SCC.	[138]

Abbreviations: 5FU—5-fluorouracil; 8-OHdG—8-oxo-2′deoxyguanosine; ABCG2—ATP binding cassette subfamily G member 2; AKR1B10 - aldo-keto reductase family 1 member B10; AKR1C3—aldo-keto reductase family 1 member C3; AML—acute myeloid leukemia; AMPK—5’-AMP-activated protein kinase catalytic subunit alpha-2; ARE—antioxidant response element; BACH1—BTB and CNC homolog 1; BCL-2—BCL2 apoptosis regulator; BCL-XL—Bcl-2-like protein 1; BTC—biliary tract cancer; CAT—catalase; CLL—chronic lymphocytic leukemia; CNA—copy number alterations; CNH—copy-number high group; CRC—colorectal cancer; CUL3—cullin 3; CXCL13—C-X-C motif chemokine 13; CXCR5—C-X-C chemokine receptor type 5; EC—endometrial carcinoma; ERα—estrogen receptor α; ESC—esophageal squamous cell carcinomas; ESCC—esophageal squamous cell carcinomas; FAM5C—BMP/retinoic acid inducible neural specific 3; GCLC—glutamate-cysteine ligase, catalytic subunit; GCLM—glutamate-cysteine ligase, modifier subunit; GPX4—glutathione peroxidase 4; GSR—glutathione reductase; GSTM1—glutathione *S*-transferase mu 1; HCC—hepatocellular carcinoma; HMOX-1—heme oxygenase 1; HNSCC—head and neck squamous cell carcinoma; IHC expression—immunohistochemical expression; KEAP1—Kelch-like ECH-associated protein 1; KRAS—Kirsten rat sarcoma viral oncogene homolog; MAGEC1—MAGE family member C1; miR—microRNA; NAMS—NFE2L2-associated molecular signature; NFE2L2—nuclear factor, erythroid 2 like 2; NF-κB—nuclear factor κB; NLRP3—NLR family pyrin domain containing 3; NQO1—NAD(P)H quinone dehydrogenase 1; NRF1—nuclear factor, erythroid 2 like 1; NRF2—nuclear factor, erythroid 2 like 2; NSCLC—non-small cell lung cancer; PARK7—Parkinson disease protein 7; PPP—the pentose phosphate pathway; RELA—RELA proto-oncogene, NF-kB subunit; ROR1—receptor tyrosine kinase like orphan receptor 1; ROS—reactive oxygen species; SCC—squamous cell carcinoma; SNP—single nucleotide polymorphism; SOD—superoxide dismutase; SRXN1—sulfiredoxin 1; TNM stage—cancer staging system with categories: Tumor, Node, Metastasis; TRIM25—tripartite motif-containing protein 25; TRX—thioredoxin; TTF1—thyroid transcription factor-1; WT—wild-type.

**Table 2:** The implication of the TRX system and TXNDC protein family in cancer.

Tumor Type	Involvement of TRX System	Ref.
Basal cell carcinoma	TRXR activity is higher in tumor tissues compared to adjacent healthy tissue.	[172]
Blood cancer	Poor survival is correlated with a lower expression of TXNIP in acute myeloid leukemia.	[162]
	The human histiocytic/monocytic leukemia cells have several-fold higher TRXR expression compared to non-transformed cells. Both normal and transformed cells were found to secrete TRXR.	[173]
	TRX is overexpressed in T-Cell acute lymphoblastic leukemia cells.	[174]
Brain cancer	Excessive cytoplasmic TRXR is correlated with a worse prognosis of brain cancer patients.	[142]
	TRX expression is positively correlated with increasing grades of glioma.	[154]
	TXNIP high expression is associated with a lower pathological grade of meningioma.	[175]
Breast cancer	TRX1 and TRXR1 are overexpressed in tumor tissue and are correlated with poor survival.	[143]
	TXNIP overexpression is correlated with better survival.	[163]
	TRXR1 overexpression is associated with the occurrence of metastasis, while TXNIP overexpression correlated with a better prognosis.	[176]
	Poor survival of triple-negative breast cancer patients correlates with high c-MYC and low TXNIP expression.	[177]
Cervical squamous cell carcinoma	High expression of TRX1 is associated with poor response to cisplatin-based neoadjuvant chemotherapy.	[178]
Cholangiocarcinoma	TRX is overexpressed in tumor tissue and in dysplastic bile ducts with highly abnormal growth patterns.	[150]
Clear cell renal cell carcinoma	TXNDC5 is overexpressed in tumor tissues compared to adjacent normal tissues.	[165]
Colorectal cancer	Thioredoxin-like protein 2 expression is increased in tumor tissues and correlates with its histological grade and prognosis.	[179]
	TRX1 is overexpressed in tumor tissues and associated with clinicopathological features and poor survival.	[155]
	TXNDC5 is overexpressed in tumor tissues.	[166]
	TXNDC9 expression is associated with tumor histological grade and survival.	[169]
Gallbladder carcinoma	TRX1 expression is higher in gallbladder carcinoma.	[151]
Gastric cancer	High TXNDC5 expression correlates with poor prognosis.	[168]
	High TXNIP and low TRX correlates with better prognosis, while low TXNIP and high TRX correlates with a poor prognosis.	[156]
	High TRX1 expression in gastric cancer tissues is associated with poor survival.	[157]
	TRXR activity is significantly higher in the plasma of gastric cancer patients compared to healthy controls.	[180]
Hepatocellular carcinoma	TRX expression is overexpressed in HCC compared to the control group.	[152]
	TRXR1 and TRX are upregulated in the tumor.	[144]
	TXNIP expression is significantly decreased in tumor tissues.	[159]
Lung cancer	High TRXR expression is associated with the poor prognosis of NSCLC patients.	[181]
	TRXR1 mRNA and protein are overexpressed in NSCLC.	[145]
	TRXR2 is upregulated in NSCLC tumor tissues.	[149]
	TXNDC5 is upregulated in NSCLC tumor tissue.	[167]
	TRX1 expression correlated with the degree of NSCLC tumor differentiation.	[182]
	TXNIP is correlated with a good prognosis of lung large-cell carcinoma patients.	[164]
	TRXR1 and TRX are upregulated in lung adenocarcinoma.	[144]
Oral squamous cell carcinoma	TRXR1 is overexpressed in oral carcinoma patients.	[146]
	TRXR1 is overexpressed in tumors and correlates with the clinical stage and metastasis.	[147]
Ovarian cancer	Nuclear TRX expression was lower in borderline tumors compared to benign ovarian epithelial tumors.	[183]
	TXNDC17 is overexpressed in tumor tissue and correlates with poor prognosis and shorter survival of patients.	[171]
Prostate cancer	Levels of TRX1 increase with cancer progression in androgen-deprived castration-resistant prostate cancer cells.	[158]
	TRX1 protein is overexpressed, but its activity unchanged, in high-grade prostate cancer compared with adjacent normal tissue.	[184]
	Tumors have increased TXNDC9, and it correlates with advanced clinical stages.	[170]
	TXNIP expression is decreased in prostate cancer.	[160]
Thyroid cancer	TRXR1 expression is decreased in thyroid cancer cells compared to healthy cells.	[185]
	TXNIP is highly expressed in differentiated thyroid cancer, while its expression is low in anaplastic thyroid cancer.	[161]
	TRX and TRXR are overexpressed in the cytoplasm and nuclei of tumor cells compared to normal tissue.	[153]
Tongue squamous cell carcinoma	TRX and TRXR1 are highly expressed in tumor tissue.	[148]
Uveal melanoma	Poor survival and metastasis are associated with the high uveal melanoma tissue expression of peroxiredoxin-3.	[186]

Abbreviations: HCC—hepatocellular carcinoma; NSCLC—non-small cell lung cancer; TRX—thioredoxin; TRXR—thioredoxin reductase; TXNDC—thioredoxin-domain containing proteins; TXNIP—thioredoxin interacting protein.

**Table 3:** Mechanisms of action of the TRX system or TXNDC protein family in tumorigenesis.

TRX System/TXNDC	Mechanism	Ref.
TRX1/2	TRX alters the function of therapeutic monoclonal antibodies by reducing the antibodies’ interchain disulfide bonds.	[219]
	Joint inhibition of TRX, GSH, and NRF2 promotes intracellular ROS and suppresses the growth of head and neck cancer cells.	[220]
	TRX phosphorylation at T100 attributes to its anti-apoptotic effects in tumor cells.	[221]
	TRX knockdown induces G1 phase cell-cycle arrest through the ERK1/2-cyclin D1 pathway.	[222]
	Nitric oxide synthase type 3 and S-nitrosation of the CD95 receptor is induced by TRX1. This results in the increased activity of caspase-8, while the activity of caspase-3 is decreased promoting the proliferation of liver cancer cells.	[187]
	TRX1 overexpression decreases PTEN; increases the amount of phosphorylated AKT; and promotes the growth, migration, and invasion of gastric cancer cells. Contrary, TRX1 silencing has the opposite effect.	[157]
	TRX1 promotes epithelial to mesenchymal transition of colorectal cancer cells through the phosphorylation of AKT, leading to the upregulation of S100A4.	[190]
	TRX1 inhibition induces intracellular ROS, elevates TP53 and androgen receptor levels, and promotes cell death. Additionally, the androgen receptor levels under androgen deprivation are increased in castration-resistant prostate cancer cells when TRX1 is inhibited.	[158]
	TRX1 plays a role in keeping mixed-lineage kinase domain-like protein, necessary for necroptosis activation, in a reduced inactive state.	[223]
	TRX1 activates the transcription of S100P, which in turn downregulates TXNIP and upregulates p-ERK1/2, thus promoting TRX1 expression in colorectal cancer cells.	[155]
	Upregulation of TRX1 induces matrix metalloproteinase 9 expression, promoting the invasion of breast cancer cells.	[224]
	Depletion of ubiquitin-like with PHD and RING finger domains 1 reduces TRX2 and increases intracellular ROS in retinoblastoma cells.	[225]
	Glioma nitric oxide synthase 2 induces the *S*-nitrosylation of TRX2 and mitochondrial caspase 3 in microglial cells, reducing their activity and promoting tumorigenesis.	[188]
TRXR1/2/3	Mitochondrial TRXR3 reduces TRX2 and stabilizes mitochondrial-associated survival molecules, thus promoting cell survival.	[191]
	TRXR inhibition alters the mitochondrial membrane and induces the apoptosis of liver cancer cells.	[192]
	TRXR inhibition promotes heme oxygenase-1 overexpression, allowing tumor cells to survive, while the simultaneous inhibition of both induces the apoptosis of myeloma cells.	[193]
	Lysine oxidase induces ROS, activates caspase-independent cell death, and promotes TRXR1 via NRF2 in triple-negative breast cancer cells.	[226]
	ROS promotes miR-526b/miR-655 expression, consequently leading to the upregulation of TRXR1 in cancer cells.	[227]
	miR-125b-5p inhibits TRXR1 in HCC cells.	[194]
	Acetylation of TRXR1 multimers promotes the formation of more active TRXR1 dimers. Additionally, acetylation of TRXR1 at Lys307 results in a2.7-fold increased catalytic activity.	[198]
	Overexpression of miR-124 binds to 3’-UTR of TRXR1 and reduces its expression.	[195]
	TRXR1 is susceptible to nitrosylation, resulting in TRXR1 inactivation.	[197]
	Upregulation of mature miR-17-3p inhibits TRXR2 and suppresses mitochondrial respiration, rendering prostate cancer cells more sensitive to ionizing radiation.	[196]
	TRXR2 inhibition promotes ROS formation; decreases the activity of SOD, CAT, and GPX1, and reduces growth; and induces the apoptosis of NSCLC cells.	[149]
TXNDC	Circular RNA, circRNA-104718, competes with TXNDC5 mRNA for miR-218-5p, and its overexpression promotes tumor growth and metastasis.	[228]
	ER stress induces the association of sulfiredoxin with TXNDC5, and, depending on the levels of each, they have a different impact on cancer patient survival.	[229]
	Inactivation of NR4A1 downregulates TXNDC5, thus promoting intracellular ROS and IL24 expression. This in turn inhibits the growth and induces apoptosis of rhabdomyosarcoma.	[214]
	TXNDC5 expression might be induced under hypoxic conditions by upregulating HIF1α and thus supporting the tumorigenesis of colorectal cancer cells.	[166]
	Inhibition of TXNDC5 promotes the expression of serpin peptidase inhibitor, clade F, and TNF receptor-associated factor 1, inducing apoptosis and inhibiting angiogenesis in cervical cancer.	[217]
	Androgen deprivation induces the hypoxia of prostate cancer cells by downregulating miR-200b, promoting HIF1α, and increasing TXNDC5, which directly interacts with the androgen receptor, promoting its stability during cancer progression.	[218]
	Inactivation of NR4A1 downregulates TXNDC5, isocitrate dehydrogenase 1, and the mTOR pathway, promoting intracellular ROS, inducing apoptosis, and inhibiting the growth of kidney cancer cells.	[215]
	Downregulation of NR4A1 downregulates TXNDC5 and isocitrate dehydrogenase 1, activates oxidative and ER stress, and inhibits the mTOR pathway in breast cancer cells.	[216]
	TXNDC9 interacts with peroxiredoxin-1 and MDM2 in prostate cancer cells. Elevated ROS induce TXNDC9 overexpression, triggering the dissociation of peroxiredoxin-1 and the degradation of MDM2, thus promoting the androgen receptor signaling, growth, and progression of prostate cancer cells.	[170]
TXNIP	Inhibition of class I histone deacetylases promotes TXNIP expression, promoting the ROS-induced DNA damage and apoptosis of BRCA1-deficient breast cancer cells.	[207]
	Overexpression of TXNIP promotes the apoptosis of prostate cancer cells and induces G0/G1 cell cycle arrest.	[160]
	Inhibition of bromodomain and extra-terminal domain downregulates MYC, leading to the upregulation of TXNIP, excessive intracellular ROS, and promoting the apoptosis of BRCA1-deficient breast cancer cell death.	[209]
	p38 mitogen-activated protein kinase phosphorylates TXNIP, predominantly at Ser361, promoting its association with JAB1 and inducing G1/S cell cycle arrest.	[230]
	c-MYC-driven glycolysis in prostate cancer cells is accomplished through the activation of glutaminolysis via glutaminase, inducing the blockage of MondoA activity and yielding the suppression of TXNIP.	[231]
	Oncogenic Ras targets the N-terminus of TXNIP, suppressing its synthesis via altered translation rate by ribosomes.	[202]
	TXNIP forms a complex with hypoxia-inducible factor 1α and mediates its nuclear export and degradation. miR-224 binds to the 3’-UTR of TXNIP, altering the nuclear export of hypoxia-inducible factor 1α and promoting the proliferation and migration of pancreatic cancer cells.	[204]
	Metabolic/oxidative stress induces TXNIP expression, while insulin-like growth factor 1 inhibits TXNIP.	[199]
	TXNIP overexpression induces ROS generation by mitochondria, activates the MAPK pathway, promotes apoptosis, and decreases the growth of HCC cells.	[159]
	Inhibition of the PI3K/AKT pathway promotes TXNIP expression, which inhibits the plasma membrane localization of glucose transporter 1 in NSCLC cells.	[210]
	c-MYC binds to an E-box-containing region of TXNIP promoter, downregulating TXNIP and leading to elevated glucose uptake in triple-negative breast cancer cells.	[177]
	Downregulation of the HER-1/2 pathway induces TXNIP expression, which further promotes the p27 expression, apoptosis, and G1 cell cycle arrest of breast cancer cells.	[163]
	TWIST acts as a transcription factor that, by binding to the miR-371-373 gene cluster promoter, upregulates miR-373 expression. MiR-373 targets 3’-UTR of TXNIP, suppressing it, which in turn induces hypoxia-inducible-factor 1α and TWIST, promoting the epithelial-to-mesenchymal transition and metastasis of breast cancer cells.	[205]
	Hypoxia induces TXNIP expression in NSCLC cells.	[200]
	Hyperglycemia induces TXNIP overexpression in pancreatic cancer cells.	[201]
	Focal adhesion kinase overexpression inhibits TXNIP expression, while its downregulation upregulates TXNIP in cancer cells.	[211]
	TXNIP inhibition upregulates the transforming growth factor-β pathway and promotes epithelial to mesenchymal transition in lung cancer cells.	[206]
	Downregulation of histone deacetylase 10 induces TXNIP expression in gastric cancer cells.	[208]
	p21WAF1 binds to the TXNIP promoter, suppressing its expression and inducing TRX and angiogenesis in breast, lung, and prostate cancer cells.	[203]

Abbreviations: 3′-UTR—3′-untranslated region; AKT—protein kinase B; BRCA1—Breast cancer susceptibility gene 1; CAT—catalase; ER—endoplasmic reticulum; ERK1/2—extracellular signal-regulated kinase 1/2; GPX—glutathione peroxidase; GSH—reduced glutathione; HCC—hepatocellular carcinoma; HIF1α—hypoxia inducible factor 1α; JAB1—Jun activation domain-binding protein-1; MAPK—mitogen-activated protein kinase; miR—microRNA; mTOR—mammalian target of rapamycin; NR4A1—nuclear receptor 4A1; NRF2—nuclear factor, erythroid 2 like 2; NSCLC—non-small cell lung cancer; PI3K—phosphatidylinositol-3-kinase; PTEN—Phosphatidylinositol 3,4,5-trisphosphate 3-phosphatase and dual-specificity protein phosphatase; ROS—reactive oxygen species; SOD—superoxide dismutase; TRX—thioredoxin; TRXR—thioredoxin reductase; TWIST—Twist basic helix-loop-helix transcription factor; TXNDC—thioredoxin-domain containing proteins.

**Table 4:** The implication of the GSH system in cancer.

Tumor Type	Involvement of the GSH System	Ref.
Bladder cancer	GPX2 is overexpressed in papillary urothelial carcinoma.	[244]
	GSTO1 expression correlates with tumor grade and stage of urinary bladder carcinoma.	[252]
Blood cancer	GPX is increased in acute myeloblastic leukemia.	[261]
	GPX4 expression correlates with the poor survival of patients with large B-cell lymphoma.	[247]
	Blood GPX level is decreased in multiple myeloma patients.	[239]
	Leukemia patients have excessive leukocyte superoxide anion generation and elevated red cell GPX and SOD activity.	[262]
	Lymphocytes of chronic lymphocytic leukemia patients have increased GPX, GSH, 8-OHdG, and lipid peroxidation, while SOD and CAT are decreased.	[263]
	GSTP1 is decreased in lymphoma.	[264]
	Downregulation of CAT, GPX, SOD, and TRX inhibitor is associated with the poor prognosis of diffuse large B-cell lymphoma patients.	[265]
Breast cancer	Breast cancer patients have a lower GPX activity in serum.	[266]
	GPX3 promoter is hypermethylated and GPX3 expression downregulated in inflammatory breast cancer tissues.	[240]
	GSTP1 hypermethylation correlates with the increased tumor grade of triple-negative breast cancer patients.	[256]
	Serum GPX activity is decreased in cancer patients compared to healthy control.	[236]
	Serum GPX activity is decreased in cancer patients.	[237]
Cervical cancer	GPX2 expression is upregulated in tumor tissue.	[245]
Colorectal cancer	GPX activity is increased in tumor tissue compared to normal tissue.	[267]
	GSH level and expression of GPX1 and GPX3 are lower in tumor tissue compared to normal tissue. On the contrary, GPX2 expression is increased.	[235]
	GSTP1, GSTT1, GSTO1, and GSTK1 expression is upregulated in tumor tissue compared to adjacent normal tissue.	[253]
Esophageal carcinoma	Serum GPX and GR activities are decreased in esophageal squamous cell carcinoma cancer patients.	[238]
	The tumor has higher GPX3 methylation and lower GPX3 activity compared to paired normal tissue.	[268]
Gastric cancer	Blood GSH is decreased in cancer patients.	[233]
	GPX2 expression is upregulated in tumor tissue and lymph node metastases.	[246]
	GPX7 is downregulated in almost 50% of gastric cancer samples.	[241]
Head and neck carcinoma	Blood GSH is decreased in cancer patients, while it is increased in tumor tissue compared to adjacent normal tissue.	[234]
Hepatocellular carcinoma	GPX4 and gamma-glutamyltransferase 1 expression is increased, while GCL, GR, GPX1, and GSS are decreased in liver tumor tissue compared to the surrounding normal tissue.	[144]
	GSTA1 expression is downregulated in HCC and correlates with poor prognosis.	[248]
	GSTM1 expression is downregulated in HCC.	[249]
	GSTZ1 expression is downregulated in HCC.	[63]
	GSTZ1 expression is downregulated in tumor tissue compared to adjacent normal tissue and correlates with poor prognosis.	[250]
	High GSTP1 expression correlates with better survival and smaller tumor size.	[254]
Lung cancer	GPX3 expression is decreased in NSCLC tissues.	[242]
	GSTP1 expression is increased while GCL and gamma-glutamyltransferase 1 are decreased in tumor tissue compared to the surrounding normal tissue.	[144]
Oral squamous cell carcinoma	GPX1 and GPX4 are overexpressed in oral carcinoma correlates with grade and stage and with poor survival.	[146]
Ovarian cancer	GPX levels are decreased in cancer patients.	[269]
	Serum GPX3 is decreased in cancer patients and correlates with the stage of the disease.	[270]
Pancreatic cancer	GPX1 expression is lower in tumor tissues compared to adjacent normal tissue and correlates with poorer prognosis.	[243]
Prostate cancer	GSTM1 expression is downregulated in prostate cancer.	[251]
	GSTP1 methylation was detected in more than 80% of tumor tissues and approximately 40% of adjacent non-neoplastic tissue.	[257]
	The incidence of GSTP1 methylation is higher in malign than in benign tissue samples.	[258]
	Plasma GSTP1 is hypermethylated in cancer patients.	[259]
	Tumor tissues have low GSTP1 expression.	[255]
	Undetectable methylated GSTP1 DNA in serum correlates with a better prognosis.	[260]
Thyroid cancer	Papillary thyroid carcinoma tissue has a higher expression of GPX7 compared to nodular goiter.	[271]

Abbreviations: 8-OHdG—8-oxo-2′deoxyguanosine; CAT—catalase; GCL—glutamate cysteine ligase; GPX—glutathione peroxidase; GR—glutathione reductase; GSH—reduced glutathione; GSS—glutathione synthetase; GST—glutathione *S*-transferase; HCC—hepatocellular carcinoma; NSCLC—non-small cell lung cancer; SOD—superoxide dismutase.

**Table 5:** Mechanisms of action of the GSH system in tumorigenesis.

GSH System	Mechanism	Ref.
GCL	NRF2 overexpression promotes the expression of GCL, elevating GSH and supporting tumorigenesis, while its downregulation elevates ROS and induces G1 cell cycle arrest and apoptosis.	[110]
	NRF2/AP-1 induces the upregulation of the GCL subunit, leading to increased GSH.	[272]
GPX	GPX4 activity is negatively regulated by acetylated high-mobility group box-1, consequently promoting inflammation.	[280]
	MiR-196a targets GPX3, downregulating its expression and promoting the tumorigenicity of NSCLC cells.	[242]
	GPX4 deficiency induces G1/G0 cell cycle arrest and inhibits tumorigenesis in pancreatic cancer stem-like cells.	[278]
	GPX2 overexpression correlates with the activation of epithelial to mesenchymal transition, the activation of β-catenin-WNT signaling, and the increased proliferation and metastasis of cervical cancer cells.	[245]
	GPX1 downregulation activates the AKT/GSK-3β/SNAIL pathway, promoting the epithelial to mesenchymal transition of pancreatic ductal adenocarcinoma cells.	[243]
	TFAP2C targets GPX1 promoter inducing GPX1 expression, while the CpG island methylation of GPX1 promoter downregulates its transcription in breast cancer.	[274]
	GPX3 interacts with TP53-induced gene 3, enhancing ROS production in prostate cancer cells. When this interaction is affected, the GPX3-mediated cell death is decreased.	[275]
	Upregulation of mature miR-17-3p inhibits GPX2 and suppresses mitochondrial respiration, rendering prostate cancer cells more sensitive to ionizing radiation.	[196]
GR	GR inhibition reduces vimentin, ERK1/2, and SNAIL transcription, while it increases the E-cadherin expression, altering the epithelial to mesenchymal transition of melanoma cells.	[281]
GSH	When GSH is depleted, protein homeostasis is maintained in cancer cells by deubiquitinases.	[273]
	Homocysteine induces NRF2, leading to increased GSH expression in liver cancer cells.	[282]
	Quinolone-indolone conjugate 2 decreases GSH.	[283]
	Nutrient deprivation promotes c-MYC expression, which upregulates the serine biosynthesis pathway, leading to increased GSH generation and supporting the survival and proliferation of tumor cells.	[284]
GST	Overexpression of piR-31470 induces GSTP1 inactivation by the methylation of CpG island.	[277]
	Long intergenic noncoding RNA 00844 recruits early B cell factor 1 to the GSTP1 promoter, inducing its expression and leading to the attenuated growth of prostate cancer.	[255]
	GSTM1 overexpression reduces ROS and elevates GSH and TP53.	[249]
	GSTZ1 downregulation reduces GSH, contributing to the promotion of oxidative stress and the constitutive activation of the KEAP1/NRF2 pathway, thus promoting cancer progression.	[250]
	GSTZ1-1 deficiency induces the accumulation of succinylacetone oncometabolite and alkylates KEAP1, leading to the activation of NRF2 signaling pathway and the transcription of insulin-like growth factor. This in turn promotes tumor growth.	[63]
	Long intergenic noncoding RNA 01419 overexpression promotes the methylation of GSTP1 in esophageal squamous cell carcinoma.	[285]
	MiR-133b targets 3’-UTR of GSTP1, downregulating its expression.	[276]
	GSTP1 overexpression upregulates p21 and p27, while it downregulates pAKT, inducing the G1/S cell cycle arrest of liver cancer cells.	[254]
	GSTA4 overexpression induces the AKT pathway, promoting the tumorigenesis of HCC.	[286]

Abbreviations: 3′-UTR—3′-untranslated region; AKT—protein kinase B; AP-1—Activator protein 1; ERK1/2—extracellular signal-regulated kinase 1/2; GCL—glutamate cysteine ligase; GPX—glutathione peroxidase; GR—glutathione reductase; GSH—reduced glutathione; GSK-3—glycogen synthase kinase-3; GST—glutathione *S*-transferase; HCC—hepatocellular carcinoma; KEAP1—Kelch-like ECH-associated protein 1; miR—microRNA; NRF2—nuclear factor, erythroid 2 like 2; ROS—reactive oxygen species; TFAP2C—Transcription factor activating enhancer-binding protein 2C.

**Table 6:** Summary of the described antioxidant defense mechanisms in cancer incidence and anticancer therapy.

Modulators	Examples
Vitamins	alpha-tocopherol
	beta-carotene cancer
Nrf2 activators	broccoli sprout extracts/sulforaphane
	curcumin
	resveratrol
	bardoxolone-methyl (CDDO-Me)
	oltipraz
	RTA-408 (omaveloxolone)
	saxagliptin and sitagliptin
Nrf2 inhibitors	all-trans retinoic acid (ATRA)
	clobetasol propionate (CP)
	apigenin
	ARE expression modulator 1 (AEM1)
	ML385
	1-(2-cyclohexylethoxy)aniline (IM3829)
	malabaricone-A (MAL-A)
TRX system inhibitor	auranofin
GSH system inhibitor	buthionine sulfoximine (BSO)
PI3K/AKT inhibitor	MK2206 (Merck)
Others	glutaminase inhibitors
	immunotherapy for patients with mutations in *NFE2L2/KEAP1*
	ARE-regulated lentiviral vector, expressing HSV-TK/GCV for suicide gene therapy
